# Mucosal blood flow in the remaining rectal stump is more affected by total than partial mesorectal excision in patients undergoing anterior resection: a key to understanding differing rates of anastomotic leakage?

**DOI:** 10.1007/s00423-021-02182-0

**Published:** 2021-05-18

**Authors:** Erik Back, Fredrik Brännström, Johan Svensson, Jörgen Rutegård, Peter Matthiessen, Markku M. Haapamäki, Martin Rutegård

**Affiliations:** 1 Department of Surgical and Perioperative Sciences, Surgery, Umeå University, SE-901 85 Umeå, Sweden; 2 Department of Surgery, Södertälje Hospital, Södertälje, Sweden; 3 Department of Statistics, Umeå School of Business, Economics and Statistics, Umeå University, Umeå, Sweden; 4 Faculty of Medicine and Health, School of Health and Medical Sciences, Örebro University, Örebro, Sweden; 5 Wallenberg Centre for Molecular Medicine, Umeå University, Umeå, Sweden

**Keywords:** Rectal cancer, Perfusion, TME, PME, Laser Doppler, Flowmetry

## Abstract

**Purpose:**

Anterior resection is the procedure of choice for tumours in the mid and upper rectum. Depending on tumour height, a total mesorectal excision (TME) or partial mesorectal excision (PME) can be performed. Low anastomoses in particular have a high risk of developing anastomotic leakage, which might be explained by blood perfusion compromise. A pilot study indicated a worse blood flow in TME patients in an open setting. The aim of this study was to further evaluate perianastomotic blood perfusion changes in relation to TME and PME in a predominantly laparoscopic context.

**Method:**

In this prospective cohort study, laser Doppler flowmetry was used to evaluate the perianastomotic colonic and rectal perfusion before and after surgery. The two surgical techniques were compared in terms of mean differences of perfusion units using a repeated measures ANOVA design, which also enabled interaction analyses between type of mesorectal excision and location of measurement. Anastomotic leakage until 90 days after surgery was reported for descriptive purposes.

**Results:**

Some 28 patients were available for analysis: 17 TME and 11 PME patients. TME patients had a reduced blood perfusion postoperatively compared to PME patients in the aboral posterior area (mean difference: −57 vs 18 perfusion units; *p* = 0.010). An interaction between mesorectal excision type and anterior/posterior location was detected at the aboral level (*p* = 0.007). Two patients developed a minor leakage, diagnosed after discharge.

**Conclusion:**

Patients operated on using TME have a decreased blood flow in the aboral posterior quadrant of the rectum postoperatively compared to patients operated on using PME. This might explain differing rates of anastomotic leakage.

**Trial registration:**

ClinicalTrials.gov Identifier: NCT02401100

## Introduction

Anterior resection is the surgery of choice for rectal cancer in the mid and upper rectum [1]. While adhering to principles of dissection in embryological planes, a total mesorectal excision (TME) or a partial mesorectal excision (PME) [2, 3] can be performed based on tumour biology, where the latter technique is considered oncologically safe only for tumours of the upper rectum. Since the adoption of TME principles, rates of anastomotic leakage have increased [4, 5]. PME is associated with a lower risk of leakage than TME, thus often obviating the need of a diverting stoma [3, 6]. The pathogenesis of anastomotic leakage is not fully understood and is considered to be multifactorial [7], though a tension-free and well-perfused anastomosis is regarded as essential. Studies imply that a disturbed rectal microcirculation is related to leakage [8], while angiography data suggest that the collateral blood flow of the mid and lower part of the rectum is less pronounced than in the upper rectum [9]. Further, leakage seems to be more prevalent in the posterior rectum [10, 11]. Recently, methods to assess blood perfusion, such as fluorescence imaging, have been used increasingly; early data are promising [12], but reproducibility might be hampered as most methods are qualitative in nature, although progress in quantitative evaluation has been made [13]. Laser Doppler flowmetry (LDF) is an established quantitative method of evaluating mucosal perfusion, with good reproducibility [14–16]; while susceptible to motion artefacts and haemodynamic changes, most of the shortcomings can be overcome with protracted measurement times and a stable application system [17]. Differences in blood flow after PME compared to TME using LDF have been investigated in a previous pilot study. In comparison to PME patients, a trend of generally decreased blood flow among patients who had undergone TME was found, as well as a significantly worsened perfusion in the posterior rectal quadrant [18]. However, that study only assessed the aboral part of the anastomosis and was conducted in patients operated on with an open approach. The current study aims to reproduce the results of the previous study in a predominantly laparoscopic/robotic setting, while further investigating microcirculation differences at both ends of the anastomosis after TME compared to PME. Our primary hypothesis was that the postoperative aboral blood perfusion reduction would be more pronounced after TME compared to PME.

## Method

### Study design

This was a single-centre prospective cohort study conducted at Umeå University Hospital, Sweden, from May 2015 to March 2018, inclusive (NCT02401100). Patients planned to undergo anterior resection as part of their treatment plan for rectal cancer or high-grade dysplasia were included in the study after informed consent. The regional ethical review board at Umeå University approved the study.

TME was primarily used on patients with a tumour in the mid to lower parts of the rectum, while PME was performed for tumours in the upper part. A diverting stoma was fashioned routinely for TME patients, while this was used selectively for PME cases. Neoadjuvant therapy was provided as determined by the local multidisciplinary team meeting. High or low ligation of the inferior mesenteric artery, mobilisation of the splenic flexure, as well as use of minimally invasive techniques were left to the discretion of the surgical team. Background information and details regarding the surgery were retrieved from the patients’ medical records. Information regarding postoperative complications was collected up to 90 days after surgery.

Blood flow measurements were performed using LDF (Moor Instruments Ltd, Millwey, Axminster, UK), an established method of assessing mucosal blood perfusion. The technology uses a probe equipped with a monochromatic laser. The reflected light from the laser is collected by a receiver. The frequency of the light returning to the probe differs depending on the velocity of red blood cells in the tissue being examined. This change in frequency is known as Doppler shift. The unit of measurement is mL/min per 100 g of tissue, hereafter defined as perfusion units [17]. The measurement sites were located using a rigid sigmoidoscope. An LDF side probe, mounted on a metal holding device to reduce tremor and increase reach as well as reproducibility, was placed at the site. The light source of the sigmoidoscope was turned off before measuring. The LDF traces were monitored continuously, thus any movement of the probe would be detected. After a stable signal was achieved, a minimum of 20 consecutive seconds of stable readings were acquired. Preoperatively, intraluminal measurements were performed after the induction of anaesthesia. The measurements were made at 2 cm above the dentate line both anteriorly and posteriorly for TME patients, considered to correspond to the intended site of anastomosis. For PME patients, the preoperative measurements were made at approximately 5 cm below the inferior tumour edge, again with the intent of measuring at the future anastomotic location. Postoperatively, with the patient still under general anaesthesia, the measurements were repeated 1 cm oral and aboral to the anastomosis. All measurements were performed by the research team, and the operating surgeons were not involved or made aware of the LDF readings. Subsequently, mean values for each measurement site were extracted and placed in a database along with other patient data.

### Statistical analysis

For each patient, four differences between the preoperative measurements and the postoperative measurements were calculated. These four differences were treated as repeated measurements defined by the combinations of the factors oral/aboral and anterior/posterior location in relation to the anastomosis. To analyse the differences, a repeated measures ANOVA design was used with the location factors as within-subject variables and mesorectal excision (TME or PME) as between-subject variables. The effect of mesorectal excision was evaluated for different levels of the factors, using estimated marginal means. Model assumptions were checked by residual analysis, and one patient was removed from the analysis due to a likely measurement error in the anterior/oral measurement. Throughout the report a significance level of 5% was used. All statistical analysis was done using IBM SPSS Statistics version 26.

## Results

### Patients

Thirty-nine patients agreed to participate in this study. Six patients were excluded based on a change of surgical plan, either pre- or intraoperatively, resulting in a non-reconstructive resection being performed. Four patients were excluded due to unavailability of research staff or measurement equipment, while one more patient was excluded in the analysis phase due to measurement error (inability to obtain stable measurements). The final patient population to be analysed comprised 28 patients, consisting of 17 and 11 patients operated on using TME and PME, respectively.

The characteristic study patient was a 66-year-old male with American Society of Anesthesiologists’ (ASA) class II. The operation was normally performed with robot-assisted laparoscopic TME for a non-irradiated stage II cancer, while the typical intraoperative bleeding was 100 ml. All TME patients received a diverting loop ileostomy whereas only two did in the PME group. All anastomoses were performed with a circular stapler. Clinical characteristics stratified by type of mesorectal excision are presented in Table 1.

**Table 1:** Baseline characteristics in 28 patients undergoing anterior resection for rectal cancer or high-grade dysplasia with available blood flow measurements

		TME (*n* = 17)	PME (*n* = 11)
Categorical variables		*N* (%)	*N* (%)
Sex	Male	8 (47)	7 (64)
	Female	9 (53)	4 (36)
Smoking	Yes	1 (6)	0 (0)
	No	9 (53)	8 (73)
	Previous	5 (29)	3 (27)
	Unknown	2 (12)	0 (0)
Preoperative radiotherapy	Yes	8 (47)	2 (18)
	No	9 (53)	9 (82)
Surgical technique	Open	3 (18)	1 (9)
	Robot-assisted	14 (82)	9 (82)
	Laparoscopy	0 (0)	1 (9)
Type of anastomosis	Side-to-end	12 (71)	1 (9)
	End-to-end	5 (29)	10 (91)
Diverting stoma	Yes	17 (100)	2 (18)
	No	0 (0)	9 (82)
ASA class	I	1 (6)	2 (14)
	II	12 (71)	8 (73)
	III	4 (24)	1 (9)
Level of tie	High	10 (59)	3 (27)
	Low	7 (41)	8 (73)
pTNM	Adenoma	1 (6)	2 (18)
	pCR	1 (6)	1 (9)
	I	3 (18)	3 (27)
	II	5 (29)	3 (27)
	III	7 (41)	2 (18)
Symptomatic leakage within 90 days	Yes	2 (12)	0 (0)
	No	15 (88)	11(100)
Continuous variables		Median (IQR)	Median (IQR)
Preoperative MAP (mm Hg)		74 (69–89)	82 (79–93)
Postoperative MAP (mm Hg)		75 (73–78)	77 (70–90)
Level of tumour, lower edge (cm)		10 (9–11)	13 (12–14)
Age (years)		64 (59–68)	72 (64–79)
Body mass index (kg/m^2^)		24.8 (22.6–30.1)	24 (21.8–28.4)
Intraoperative bleeding (ml)		150 (100–250)	50 (50–100)

*TME*, total mesorectal excision; *PME*, partial mesorectal excision; *ASA class*, American Society of Anesthesiologists’ classification; *Level of tie*, high or low ligation of the inferior mesenteric artery; *MAP*, mean arterial pressure; *pCR*, pathological complete response. Percentages may not add up due to missing data

Of the 28 patients, two (7.1%) TME patients developed anastomotic leakage within 90 days after surgery. One patient was a previous smoker, while one had received preoperative radiotherapy. Neither had adverse intraoperative events reported.

### Blood flow measurements

Preoperatively, the highest mean blood flow was seen posteriorly for TME and anteriorly for PME patients. Postoperatively, patients in the TME group showed perfusion reduction in all locations, whereas the PME group had a minor increase in the anterior oral and posterior aboral area (Table 2). When comparing TME to PME patients, the TME group had a larger perfusion decrease in all areas, except in the anterior aboral area. Comparisons reached statistical significance in the posterior aboral area (−57 vs 18 perfusion units, *p* = 0.010).

**Table 2:** Blood flow measurements in perfusion units with mean differences, stratified for type of mesorectal excision and different measurement locations

Perfusion units		Preoperative	Postoperative	Difference
TME group (*n* = 17)		Mean (SD)	Mean (SD)	Mean (SD)
Oral	Anterior	93 (54)	63 (37)	−30 (59)
	Posterior	143 (61)	87 (43)	−55 (73)
Aboral	Anterior	93 (54)	84 (39)	−9 (70)
	Posterior	143 (61)	86 (62)	−57 (71)
PME group (*n* = 11)		Mean (SD)	Mean (SD)	Mean (SD)
Oral	Anterior	122 (49)	123 (59)	2 (62)
	Posterior	98 (54)	96(82)	−2 (106)
Aboral	Anterior	122 (49)	84 (48)	−37 (62)
	Posterior	98 (54)	116 (81)	18 (2)
TME vs PME				*p* value*
Oral	Anterior			0.180
	Posterior			0.124
Aboral	Anterior			0.291
	Posterior			0.010

*Repeated measures ANOVA analysis. *TME*, total mesorectal excision; *PME*, partial mesorectal excision; *SD*, standard deviation; *TME*, total mesorectal excision; *PME*, partial mesorectal excision, *Oral/Aboral Preoperative*, measurement made preoperatively 2 cm above the dentate line and at intended site of anastomosis for TME and PME patients, respectively; *Oral Postoperative*, measurement made 1 cm above the site of anastomosis; *Aboral Postoperative*; measurement made 1 cm below the site of anastomosis

**Fig. 1:**
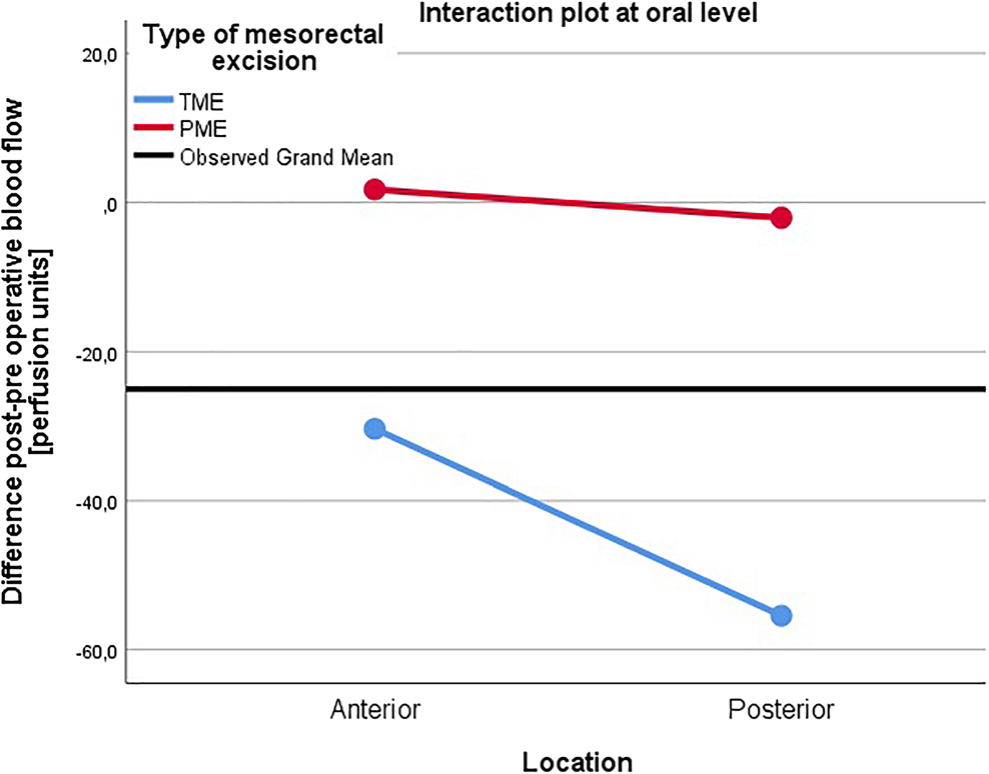
Interaction between anteroposterior location and type of mesorectal excision at the oral, evaluated in the repeated measures ANOVA model (*p* = 0.508). TME, total mesorectal excision; PME, partial mesorectal excision; PU, perfusion units

**Fig. 2:**
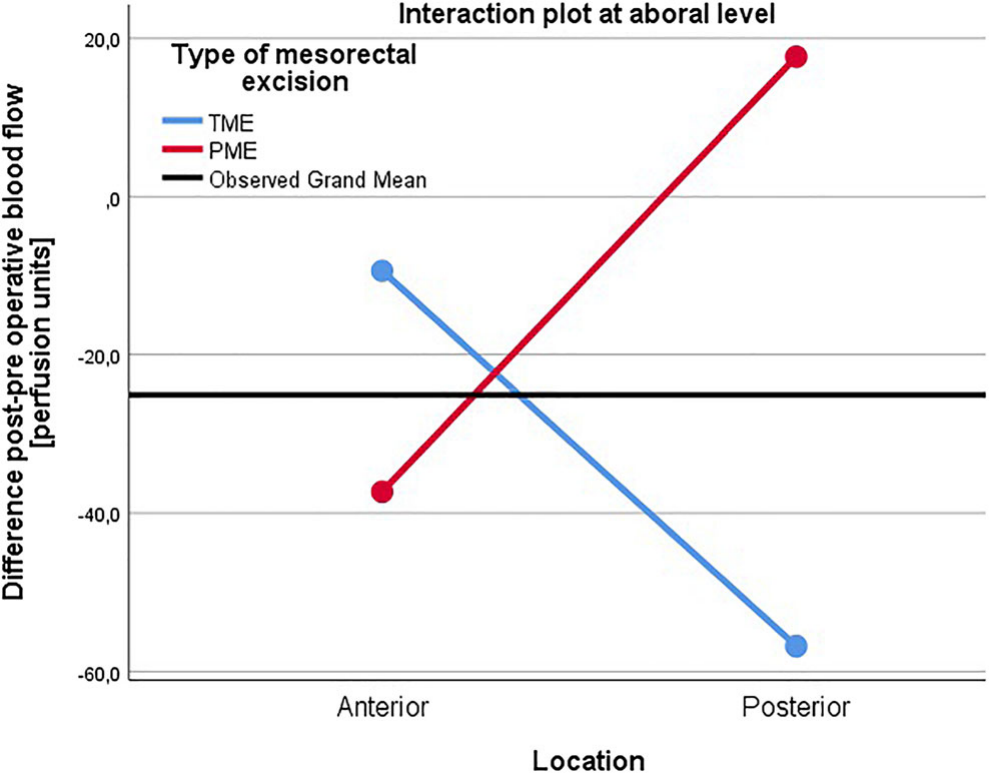
Interaction between anteroposterior location and type of mesorectal excision at the aboral level, evaluated in the repeated measures ANOVA model (*p* = 0.007). TME, total mesorectal excision; PME, partial mesorectal excision; PU, perfusion units

Using the repeated measures ANOVA design, interactions between measurement areas and type of mesorectal excision were evaluated. A significant interaction was found between type of mesorectal excision and the anterior/posterior and oral/aboral measurements (*p* = 0.005) for the entire cohort. As this implied that the type of mesorectal excision affects the interplay between the other factors to an extent where it effects perfusion changes, a more specific analysis of interaction at different measurement sites was conducted. A significant interaction occurred between mesorectal excision and anterior/posterior location at the aboral level (*p* = 0.007), whereas at the oral level it did not (*p* = 0.508). As shown in Figs. 1 and 2, the interaction in the aboral area results in a worsened blood perfusion in the posterior wall for TME patients compared to PME patients and vice versa in the anterior wall.

## Discussion and conclusions

After anterior resection, we found that there was a significantly less detrimental effect on blood flow in the posterior aboral area of the anastomosis in PME compared to TME patients, as evidenced by reductions in terms of blood flow differences before and after surgery. These findings are concordant with those of a previous pilot study [18]. We also found a significant interaction between the variables of perfusion changes for the different locations and mesorectal excision. At the aboral level, there was a larger mean perfusion reduction in the posterior compared to the anterior location for TME patients; for PME patients, there was a larger mean perfusion reduction in the anterior compared to the posterior location. At the oral level, there was a larger mean perfusion reduction in the posterior compared to the anterior location for both TME and PME patients. This could be shown statistically as a significant interaction between the anterior/posterior location and type of mesorectal excision at the aboral level, while there was no such interaction at the oral level (Figs. 1 and 2). This interaction suggests that the aboral area might be a key factor in relation to type of mesorectal excision and the resulting blood flow.

The choice to use preoperative measurements at the site of anastomosis for both TME and PME populations, rather than the same anatomical landmark for both, was due to the focus of the study being the circulation surrounding the anastomosis. However, this created a possibility of measuring at different sites for the two groups, thus posing a problem as the lower rectum has a more sparse perfusion compared to the more vascularised mid and upper parts [9]. In the PME cases, we aimed to measure at 5 cm below the inferior tumour margin; the validity of this approach was supported by the pathological examination, which showed a mean distal margin of 5.2 cm (standard deviation 2.4), in this group. It appears that the preoperative measurements indicated a higher blood perfusion in the lower part of the rectum in this study. Contrary to these findings, one study found that there was no difference in blood perfusion in the rectum with regard to the level of measurement [15]. However, in that study, measurements were not made as close to the anal verge as in our study. Further, the difference found in the present study could be explained by the proximity to the anal canal providing some stability, as the probe is sensitive to motion artefacts. It could also be argued that the preoperative rectal measurements do not correspond to the true oral part of the anastomotic site, as this would be at the colonic limb; this choice of measurement location was done as blood flow along the distal colorectum does not differ according to previous research [15], and also because the true oral transection site is impossible to determine preoperatively. The LDF methodology has other drawbacks, as it only provides a mean of the perfusion in the area with probe contact and the probe itself might also cause disturbances as it requires direct contact for measurements [17]. It is also possible that factors such as drug administration and cardiovascular status might affect blood perfusion, which could possibly alter the local haemodynamics to either create an illusion of change in perfusion, or mask an actual change [19, 20]. One study strength is that all patients were operated on at a single centre, by a small number of surgeons; the measurements were also done by a limited number of researchers, restricting the amount of people involved in the study and thus improving internal validity. Due to the small cohort size, we were not able to perform adjustments for possible confounders. Nevertheless, we analysed mean perfusion differences rather than the average perfusion before and after surgery. This made each patient her own control; confounding from patient-related factors was therefore eliminated, under the assumption that such confounding was additive rather than multiplicative. Effects of possible intraoperative technical differences between the two types of mesorectal excision, such as angle of stapling lines and multiple stapler firings, were not considered in this study. One such potential difference of importance might be the choice of vascular ligation, with high tie being more common in the TME group; this could be reflected in the seemingly larger blood flow reductions at the oral part of the anastomotic site. However, these differences did not reach statistical significance, and high ligation has neither consistently been shown to decrease blood flow of the colonic limb [18, 19], nor increase anastomotic leakage [21].

Studies using other techniques than LDF, such as fluorescent imaging, have shown promising results, with a reduced rate of leakage for patients undergoing anterior resection when applied compared to when not [12]. A recent randomised trial has been conducted, where diminished leak rates were shown in the imaging arm, though not reaching statistical significance [22]. Unlike LDF, these techniques are at the present moment qualitative rather than quantitative, rendering reproducibility a problem; as such, the technique is also difficult to use in a study on pathophysiology. In a previous report using LDF, microcirculation changes were analysed in rectosigmoid cancer surgery. The authors found a deterioration in both colonic and rectal perfusion, while the rectal stump perfusion decrease was more predictive of anastomotic leakage [8]. While this noticeable rectal microcirculation decrease was also found in the current study, the above study did not differentiate between TME and PME surgery. Interestingly, it was shown in an angiographic study that the lower rectum has a less developed arterial collateral network compared to the upper part. Of note, the authors showed that the posterior circulation in the aboral rectum had higher vascular resistance than the anterior part, implying that the former might be hypoperfused after construction of an anastomosis [9]. Further, a recent cadaver study found that the often-ignored median sacral artery might be important in relation to type of mesorectal excision. An anatomical variation in the trajectory of this artery was shown, where some patients exhibited a median sacral artery that would be cut in TME but not PME surgery, as the artery leaves the endopelvic fascia to penetrate the rectum at different heights [23]. This finding might explain parts of the herein shown link between type of mesorectal excision and reduced posterior blood flow, which in turn could be key to understanding the established elevated risk of anastomotic leakage in TME compared to PME. The proposed posterior rectal hypoperfusion is also of interest regardless of mesorectal excision type, as the posterior area of the anastomosis seems more prone to leakage than the anterior area, corroborated by clinical and radiological findings [10, 11]. However, a direct link between blood perfusion and leakage was not possible to evaluate with the limited data at hand.

In conclusion, this study was able to show a statistically significant difference in rectal blood flow between the two operating methods TME and PME in terms of mean perfusion unit differences, postoperatively compared to preoperatively, in the posterior aboral area of the anastomosis. Further research is needed to establish the potential connection between type of mesorectal excision, blood flow reduction, and subsequent anastomotic leakage.
